# Dysregulated glycosaminoglycan biosynthesis and retinoid metabolism in chronic rhinosinusitis with nasal polyps: insights from a comprehensive transcriptomic analysis

**DOI:** 10.3389/fimmu.2026.1757132

**Published:** 2026-03-23

**Authors:** Nelli Nepp, József Kun, Péter Urbán, Krisztina Pohóczky, Norbert Tóth, Krisztián Katona, Zsuzsanna Helyes

**Affiliations:** 1 Department of Otorhinolaryngology, Clinical Centre, University of Pécs, Pécs, Hungary; 2 Department of Pharmacology and Pharmacotherapy, Medical School, University of Pécs, Pécs, Hungary; 3 Omics Centre, Szentágothai Research Centre, University of Pécs, Pécs, Hungary; 4 Hungarian Research Network (HUN-REN) National Laboratory for Drug Research and Development, Budapest, Hungary; 5 Department of Dentistry, Oral and Maxillofacial Surgery, Medical School, University of Pécs, Pécs, Hungary; 6 Hungarian Research Network - University of Pécs (HUN-REN–PTE) Chronic Pain Research Group, Pécs, Hungary

**Keywords:** bioinfromatics analysis, CRSwNP, glucosaminoglycans, retinoid metabolism, RNA seq, transcriptomic analysis

## Abstract

**Background:**

Chronic rhinosinusitis with nasal polyps (CRSwNP) is a common inflammatory condition affecting 5–12% of the population. Despite its prevalence, the underlying pathophysiological mechanisms remain incompletely understood, representing an unmet medical need. Next-generation sequencing coupled with bioinformatic analysis allows to characterize the global transcriptomic profile, identify key mechanisms, pathways, and potential novel drug targets.

**Methods:**

mRNA sequencing was performed on 16 nasal polyps (CRSwNP-NP) and paired nasal mucosa (CRSwNP-NM) samples from patients with recurrent CRSwNP and on 15 nasal mucosa samples from non-CRS controls (CS-NM). Differentially expressed genes (DEGs) were determined, and enrichment analyses were performed using the Gene Ontology, Kyoto Encyclopedia of Genes and Genomes, Reactome databases and Ingenuity Pathway Analysis.

**Results:**

CRSwNP-NP tissues were differentiated from CS-NM by 183 DEGs and from CRSwNP-NM by 293 DEGs. When comparing nasal mucosa from CRS and non-CRS patients, 192 DEGs were identified. Enrichment analysis of nasal polyp tissues revealed that the most significantly upregulated gene sets were involved in positive regulation of extracellular signal-regulated kinase 1/2 cascade, hypoxia-inducible factor-1 pathway, overactivation of renin-angiotensin-aldosterone system and ferroptosis. Conversely, the downregulated gene sets were predominantly associated with impaired antimicrobial functions. Comparing patients and healthy controls’ nasal mucosal sample, glycan metabolism was significantly upregulated, while retinol metabolism was downregulated.

**Conclusion:**

The main pathways in the polyps are associated with tissue remodeling, increased renin-angiotensin-aldosterone system activation, ferroptosis, decreased antimicrobial defense as local microenvironmental risk factors contributing to the recurrence of CRSwNP. Upregulated glycosaminoglycan biosynthesis and downregulated retinol metabolism could represent a systemic susceptibility factor.

## Introduction

Chronic rhinosinusitis (CRS) is a common inflammatory disease with a complex etiology (1). CRS is classified into two distinct clinical phenotypes: CRS with nasal polyps (CRSwNP) and CRS without nasal polyps (CRSsNP). These phenotypes, however, do not predict the prognostic features or treatment outcomes of the disease (1, 2). CRS is further subclassified into several endotypes based on its underlying cellular and molecular mechanisms (3). CRS is thought to be a polygenic disorder, resulting from complex interactions between multiple genes and environmental factors. Despite significant research efforts, the precise etiology and pathogenesis remain poorly understood. Since therapy-refractory patients have diminished quality of life, CRSwNP continues to represent an unmet medical need, necessitating the precise understanding of the pathophysiological mechanisms and developing personalized therapeutic strategies (4).

Studying the manifestation of the local microenvironmental and systemic risk factors at the mRNA level in CRSwNP can provide deeper understanding of its pathogenesis, serve as prognostic biomarkers, and support the development of novel targeted therapies. Furthermore, a shift in disease management philosophy—from symptom treatment to disease modification—may become reality soon, which is also inconceivable without transcriptomic-scale investigations.

Transcriptomics and bioinformatic analysis have emerged as promising tools for screening gene expression alterations involved in the development and progression of diseases. Next generation sequencing (NGS) has rapidly developed to provide a high-throughput tool for exploring key mechanisms, mediators and targets in several conditions (5–9).

Few exploratory studies using whole transcriptome sequencing of nasal polyp tissues have demonstrated increased inflammation, impaired host defense responses, abnormal extracellular matrix (ECM) metabolism, and hemostasis. These factors can lead to the destruction of the sinonasal epithelial cell barrier and tissue remodeling (8, 10–12). However, these studies have primarily focused on eosinophilia-related mechanisms and have not addressed therapy-resistant, recurrent conditions. Furthermore, most of these studies were conducted in Asian populations and predominantly used a single control group.

In this study NGS and bioinformatic analysis were performed on polyp and nasal mucosal tissues of patients with recurrent CRSwNP patients and nasal tissue samples of non-CRS controls. These three-way comparisons using dual controls help to elucidate both systemic susceptibility and local microenvironmental factors that drive the development of CRSwNP.

## Materials and methods

### Study design and sample collection

The research was conducted at a tertiary care center, the Otolaryngology Department of the University Pécs Medical School, Hungary. It was approved by the ethics committee of the Clinical Centre of the University of Pécs (no 8555 – PTE 2020). All patients provided written informed consent for the storage of their tissue samples for research purposes.

**Figure 1:**
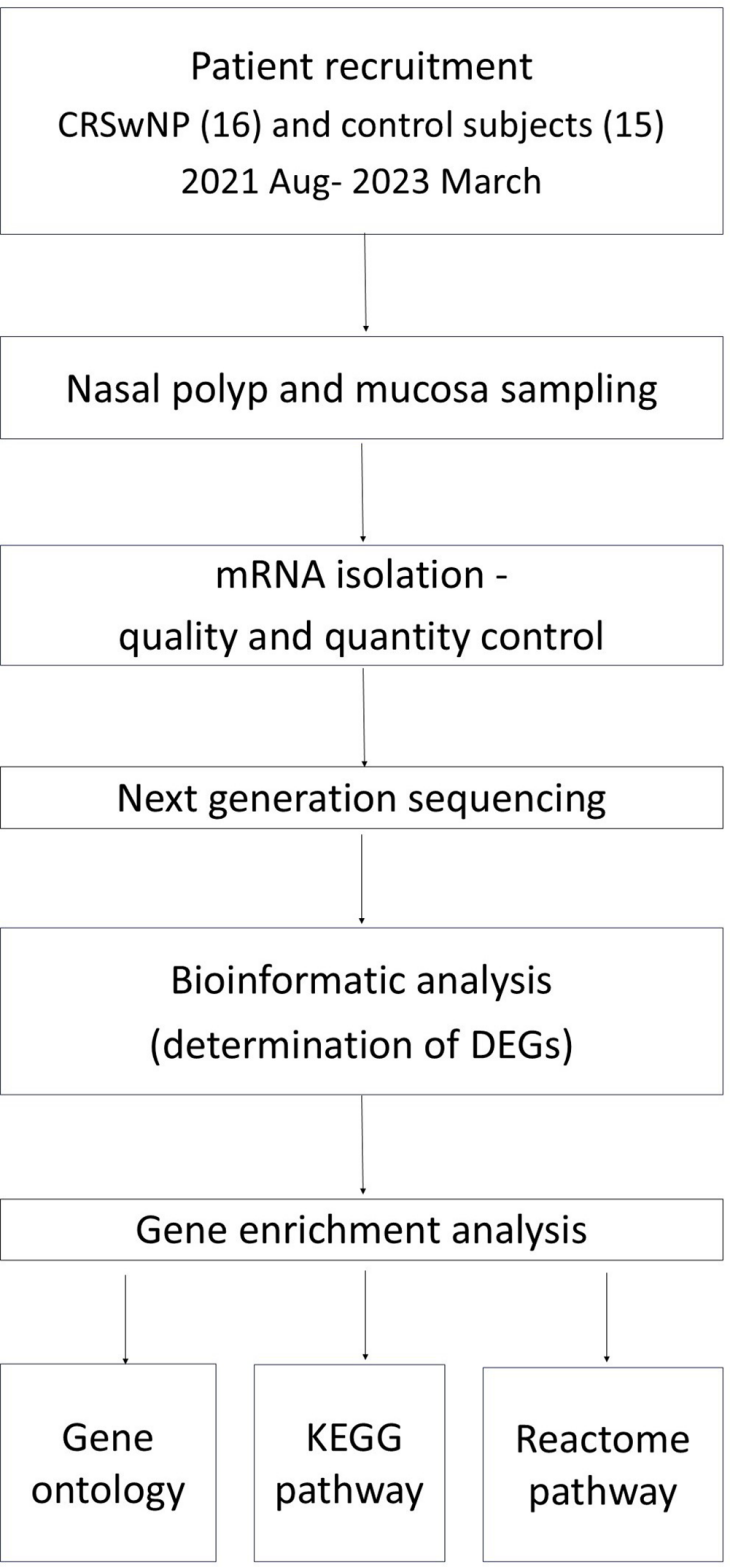
Study workflow. Study workflow from subject recruitment and sample collection to data analysis and interpretation. CRSwNP, chronic rhinosinusitis with nasal polyps; mRNA, messenger RNA; DEG, differentially expressed gene; KEGG, Kyoto Encyclopedia of Genes and Genomes.

The study protocol and investigational techniques were summarized in **Figure 1**.

Inclusion criteria included the presence of at least one previous endoscopic sinus surgery, recurrent nasal polyposis with a severity score of ≥4 on the Nasal Polyp Score (NPS, with a minimum score of 2–2 on each side) (13), only adult patients (>18 years) were recruited. CRSwNP was diagnosed according to the European Position Paper on Rhinosinusitis and Nasal Polyps 2020 (1). Patients with immunodeficiency, those undergoing biological, oral corticosteroid or other immunomodulatory therapy in the last 6 months were excluded. Control patients were selected from among adults who underwent powered endoscopic turbinoplasty (PET), dacryocystorhinostomy (DCR), or endoscopic transnasal pituitary adenoma removal, without history of CRS.

Participants were enrolled between August 2021 and March 2023. Clinical data was collected from an institutional database. Patient history was recorded, including the date of CRSwNP diagnosis, the number of previous endoscopic surgeries, the presence of asthma, aspirin-exacerbated respiratory disease (AERD), allergic rhinitis, atopic dermatitis, gastroesophageal reflux disease (GERD), and current smoking. All participants underwent nasal endoscopy. For CRSwNP patients, a CT examination was conducted prior to surgery, and the findings were categorized according to the Lund-Mackay scoring system (14). To subjectively quantify symptoms, the validated Hungarian version of the Sinonasal Outcome Test (SNOT-22) questionnaire was administered (15).

All samples were collected intra-operatively under endoscopic visualization. For CRSwNP patients, two types of tissue were obtained: polypoid mucosa from the common nasal or middle cavity (hereafter referred to as “CRSwNP-NP”) and control mucosal tissue anterior to the uncinate process (hereafter referred to as “CRSwNP-NM). Mucosal samples were obtained from control subjects (hereafter referred to as “CS-NM”) from the inferior turbinate, the covering mucosa of the lacrimal bone or the anterior wall of the sphenoid sinus, depending on the type of surgery. Following biopsy, tissues were immediately split and stored for RNA sequencing in RNA later and for histological assessment in formalin. Local eosinophilia was assessed from hematoxylin-eosin stained slides, counted the average value from 5 random high-power field, with a >10 cutoff value for local eosinophilia (16). Blood eosinophil count was also evaluated from laboratory tests, with a 0.30x10^9^/L cutoff value for systemic eosinophilia (17).

### RNA isolation

Samples were placed into 500 μL TRI‐Reagent (Thermo Fischer Scientific, Waltham, MA, USA) and homogenized with surgical scissors and Fisherbrand™ Pellet Pestle™ hand mortar (Thermo Fisher Scientific, Waltham, MA, USA). Total RNA was isolated using Direct‐zol™ RNA MiniPrep (for tissue samples bigger than 0, 5 mm) and MicroPrep (for tissue samples smaller than 0, 5 mm, Zymo Research, Irvine, CA, USA), according to the manufacturer’s instructions. The amount and purity of RNA were determined using a Jenway™ Genova Nano Micro-volume Spectrophotometer (Thermo Fisher Scientific, Waltham, MA, USA). Samples were treated with 1U DNase I enzyme to eliminate remaining genomic DNA.

### RNA sequencing

RNA libraries were generated using the QuantSeq 3’ mRNA-Seq Library Prep Kit FWD for Illumina (Lexogen, Vienna, Austria). 100 ng of total RNA was used as input for first strand cDNA generation using oligodT primer followed by RNA removing. The second strand synthesis was initiated by random priming and the products were purified with magnetic beads. The libraries were amplified and barcoded using PCR. All libraries were assessed on the Agilent 4200 TapeStation (Agilent Technologies, Palo Alto, CA, USA) to examine if adapter dimers formed during PCR. Illumina sequencing was performed on the NovaSeq 6000 instrument (Illumina, San Diego, CA, USA) with 1 × 75 run configuration.

### Bioinformatic and statistical analysis

Binary Base Call (BCL) files generated by the sequencing instrument were base called, demultiplexed and translated into FASTQ files using bcl2fastq v2.20.0.422 software (Illumina Inc.). Reads were filtered for a minimum length of 40bp and quality-trimmed to Q30 using the Phred algorithm with BBDuk (BBTools suite v38.86) (18). Reads were aligned against the human genome (GRCh37 Ensembl) with STAR v2.7.6a (19). Reads aligned within each gene were counted (HTSeq v0.11.1) (20), normalized using the trimmed mean of M values (TMM) method (edgeR v3.28, R v3.6.0, Bioconductor v3.9) and log transformed (voom approach). Fold change (FC) values and moderated t-test p-values were calculated by the limma package (21). Normalized counts were visualized as transcripts per million (TPM). Over-representation (Fisher’s exact test for Gene Ontology (GO), hypergeometric test for Kyoto Encyclopedia of Genes and Genomes (KEGG) and Reactome) and gene set enrichment analysis (non-parametric Kolmogorov-Smirnov test for GO and KEGG, hypergeometric test for Reactome) were performed (topGO v2.37.0, ReactomePA v1.30.0, gage v2.36.0). Data were mapped to KEGG pathways (pathview v1.26.0). Ingenuity Pathway Analysis (IPA) software (v111725566, QIAGEN, Venlo, Netherlands) was used for implicated canonical pathways, regulatory networks, and upstream regulators.

## Results

### Demographics of subjects

Transcriptomic analysis was performed on 16 CRSwNP patients and 15 control subjects. There were no significant differences between the two groups in terms of age and sex (p-value = 1, Fisher’s exact test of independence). However, patients with CRS had significantly higher incidence of asthma (p=0.0028) and allergic rhinitis (p=0.0180). There was no significant difference in GERD, or the proportion of current smokers. Atopic dermatitis did not occur among the participants. The CRS group also showed predominant systemic eosinophilia (75%) with a mean of 0, 41x10^9^/L blood eosinophil level, while tissue eosinophilia was present in 60% of patients, with a mean of 17, 4 in HPF. We found significantly worse quality of life in patients with CRSwNP regarding the SNOT-22 results, with an average of 54.7 points, indicating severe condition (22). 62, 5% of patients had severe, 37, 5% moderate scores. Demographics and clinical characteristics of the subjects are outlined in **Table 1**.

**Table 1:** Demographics and clinical characteristics of the subjects.

	Patients with CRSwNP	Controls
Subjects, n (%)	16 (52)	15 (48)
Age (mean., SD, min-max; years)	51.1 ± 20, 1 (18-81)	60.7 ± 13, 3 (31-81)
Females, n (%)	8 (50)	8 (53, 3)
Current smoker, n (%)	3 (18, 8)	2 (13, 3)
Asthma, n (%)	9 (56, 3)	1(6, 7)
AERD, n (%)	2 (12, 5)	0
Allergic rhinitis, n (%)	8 (50)	1(6, 7)
GERD, n (%)	1 (6, 3)	2 (13, 3)
Patients with eosinophilia in tissue samples, n (%)	8/16 (50)	0/15 (0)
Patients with eosinophilia in blood, n (%)	12/16 (75)	2/15 (13, 3)
No. of prior FESS (mean, SD, min-max)	2, 2 ± 1, 7 (1-6)	0
Lund-Mackay score (range 0-24, mean, SD, min-max)	19, 5± 3, 6(10-24)	–
Nasal Polyp Score (range 0-8, mean, SD, min-max)	5, 3 ± 1, 6 (4-8)	–
SNOT-22 score (range 0-110, mean, SD, min-max)	54, 7 ± 16, 1 (25-89)	5, 0 ± 2, 0 (0-16)

AERD, Aspirin-exacerbated respiratory disease; CRSwNP, chronic rhinosinusitis with nasal polyps; FESS, functional endoscopic sinus surgery; GERD, gastroesophageal reflux disease; SNOT, Sinonasal Outcome Test.

### Differentially expressed genes in nasal polyps of recurrent CRS patients compared to their self-control and non-CRS nasal mucosa samples

**Figure 2:**
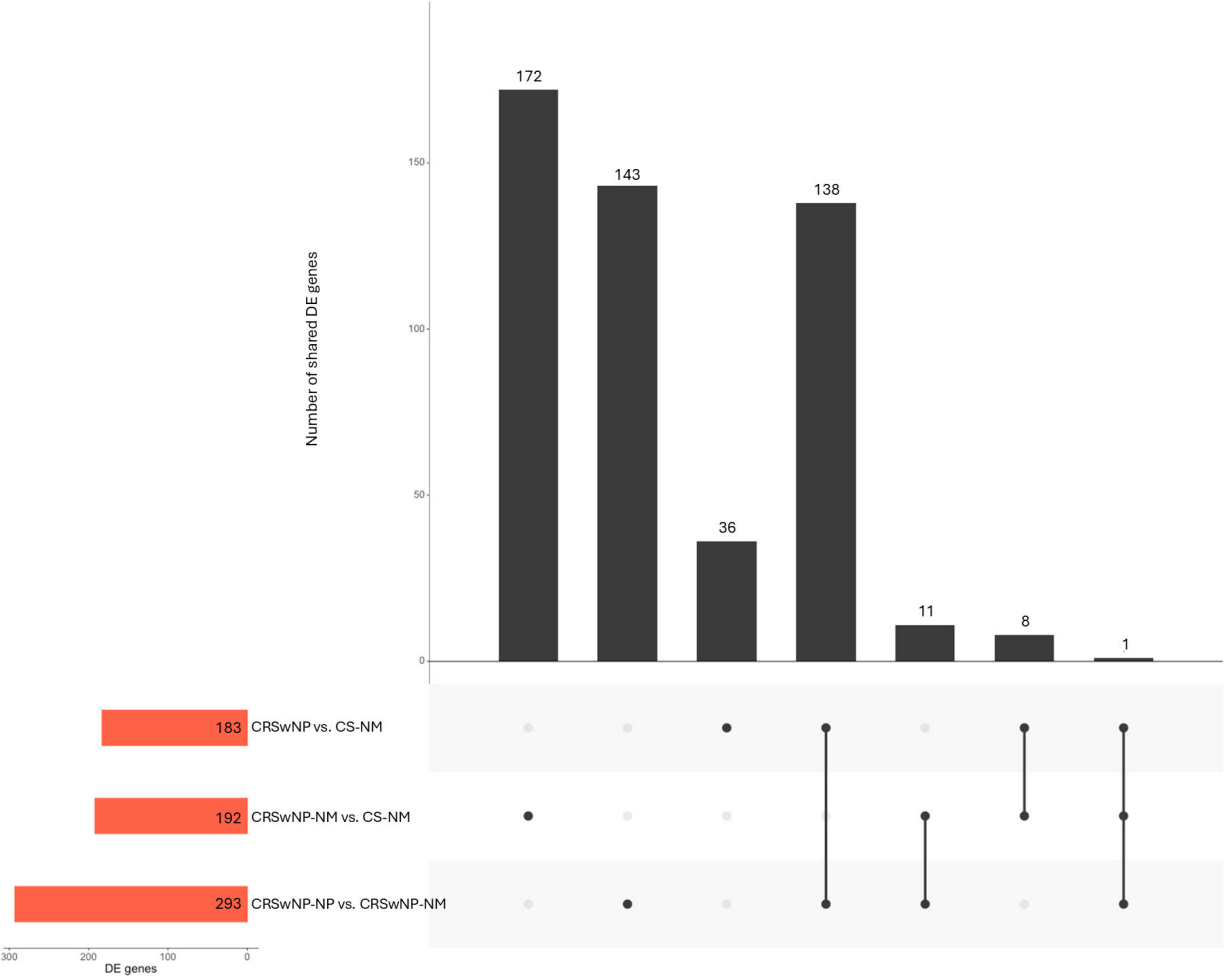
Diagram of shared differentially expressed genes in the comparisons of nasal polyps from CRS patients with recurrent disease to their self-controls and non-CRS nasal mucosal samples (colored). Horizontal red bars represent the number of DEGs in each comparison. Vertical black bars: the first three from the left depict the number of overlapping DEGs between pairwise comparisons. CRS, chronic rhinosinusitis; CRSwNP-NM, nasal mucosa from patients with chronic rhinosinusitis with nasal polyps; CRSwNP-NP, nasal polyps from patients with chronic rhinosinusitis with nasal polyps; CS-NM, nasal mucosa from control subject; DEG, differentially expressed gene.

**Figure 3:**
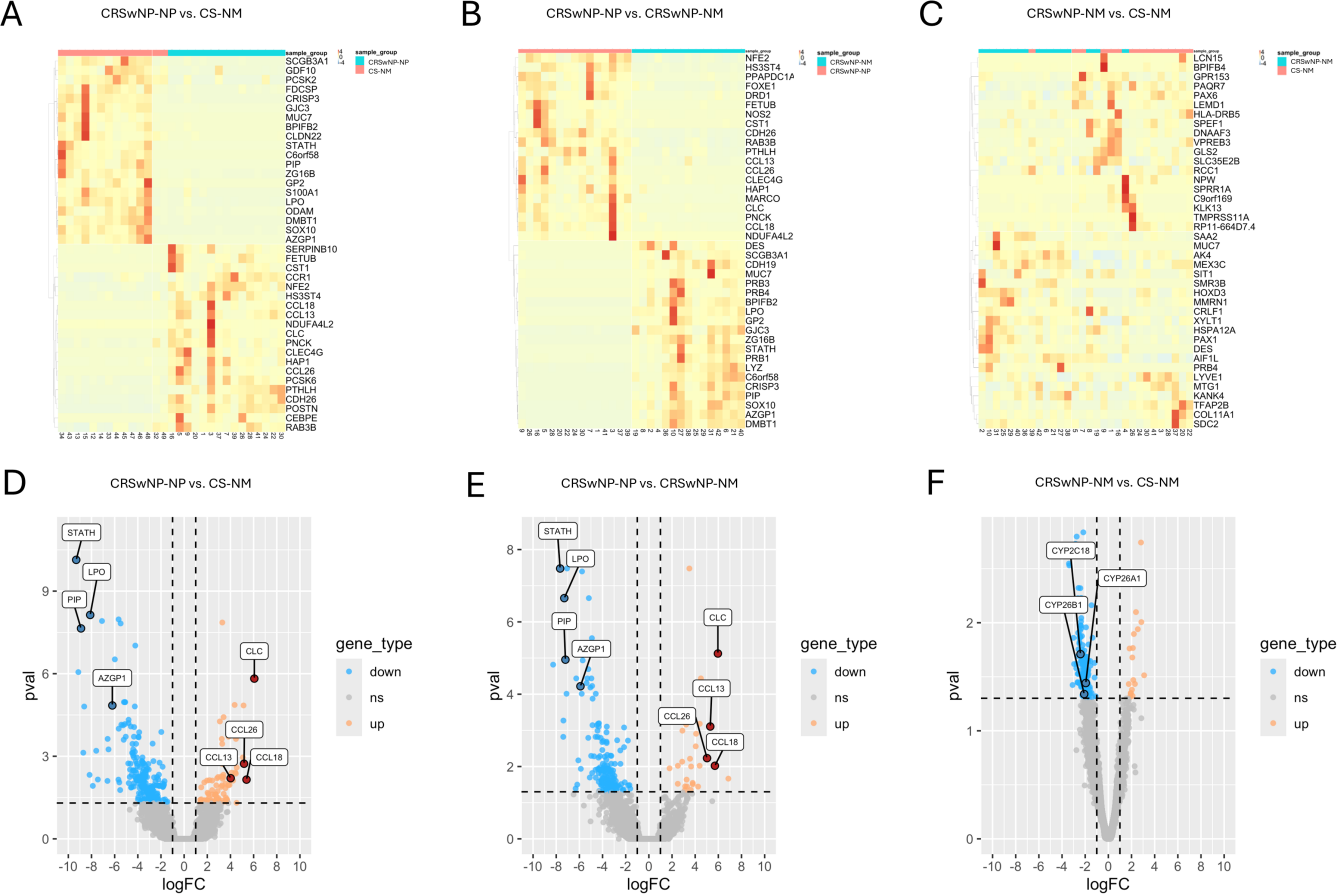
Heatmaps **(A–C)** and volcano plots **(D–F)** of DEGs in the comparisons of nasal polyps from recurrent CRS patients to their self-controls and non-CRS nasal mucosa samples (colored) Heatmaps show the top 20 upregulated (positive log fold change) and top 20 downregulated (negative log fold change) genes. In the volcano plots, each dot represents one DEG: upregulated genes are shown in red, and downregulated genes are shown in blue. CRSwNP-NM, nasal mucosa from patients with chronic rhinosinusitis with nasal polyps; CRSwNP-NP, nasal polyps from patients with chronic rhinosinusitis with nasal polyps; CS-NM, nasal mucosa from control subject; DEG, differentially expressed gene.

After processing the raw dataset, DEGs were identified and evaluated for pairwise comparison among the three tissue sample groups (CRSwNP-NP, CRSwNP-NM, CS-NM) using hierarchical clustering (**Figure 2**). The top 20 upregulated and downregulated DEGs, ranked by fold change, are presented in all comparisons in **Figure 3**. Nasal polyp tissues clearly separated from both control groups, 183 DEGs identified during CRSwNP-NP and CS-NM comparison, 293 DEGs in the CRSwNP-NP versus CRSwNP-NM comparison. In both comparisons the most upregulated genes were Charcot-Leyden crystal galectin (CLC) and C-C motif chemokine ligand 18 (CCL18), followed by Cystatin SN (CST1), CCL16 and C-Type Lectin Domain Family 4, Member G (CLEC4G). While the most downregulated ones found to be statherin and chromosome 6 open reading frame 58 (C6orf58), followed by prolactin-induced protein (PIP), lactoperoxidase (LPO) and mucin 7 (MUC7). These genes are suggested as potential polyp-specific local gene signatures. In the comparison between CRSwNP-NM and CS-NM, 192 DEGs were identified, with serum amyloid A2 (SAA2) and proline-rich protein BstNI subfamily 4 (PRB4) being the most upregulated, and major histocompatibility complex, class II, DR beta 5 (HLA-DRB5) and transmembrane protease, serine 11A (TMPRSS11A) the most downregulated.

Among all subgroup analyses, only one DEG was shared: MUC7, which was downregulated in nasal polyps compared to both control groups but upregulated in CRS nasal mucosa compared to non-CRS nasal mucosa.

### Pathophysiological pathways potentially involved in recurrent CRSwNP development determined by GO analysis

During CRSwNP-NP and CS-NM comparison, top upregulated GO sets enriched by DEGs are involved in monocyte chemotaxis, chemokine activity and receptor-binding, chemokine-mediated signaling pathway and positive regulation of extracellular signal-regulated kinase 1/2 (ERK1/ERK2) cascade. Comparing nasal polyp and nasal mucosa samples from the same patients upregulated genes are involved in response to interleukin-13 (IL-13), eosinophil chemotaxis and migration, cytokine activity. The most downregulated sets in both comparisons are related to chemical stimulus detection in sensory perception of bitter taste and antibacterial humoral response. Comparison of CRSwNP-NM and CS-NM mostly downregulated, affecting fat-soluble vitamin, arachidonic, and retinoic acid metabolic processes. Significant results from all comparisons are summarized in **Table 2**.

**Table 2:** Gene ontology analysis of biological and functional transcriptomic alterations in the pairwise comparison of nasal polyps from CRS patients with recurrent disease to their self-controls and to non-CRS nasal mucosal samples.

GO identifier	Description	Significant/annotated n	Expected n	P-value	Ontology
Upregulated					
CRSwNP-NP vs CS-NM					
GO:0002548	monocyte chemotaxis	4/54	0, 12	1, 2x10^-6^	BP
GO:0070098	chemokine-mediated signaling pathway	4/69	0, 15	1, 3x10^-5^	BP
GO:0070374	positive regulation of ERK1 and ERK2 cascade	5/157	0, 34	1, 8x10^-5^	BP
GO:1990868	response to chemokine	4/76	0, 16	1, 9x10^-5^	BP
GO:0034341	response to interferon-gamma	4/108	0, 23	7, 6x10^-5^	BP
GO:0070372	regulation of ERK1 and ERK2 cascade	5/224	0, 48	9, 9x10^-5^	BP
GO:0070371	ERK1 and ERK2 cascade	5/244	0, 52	1, 5x10^-4^	BP
GO:0048020	CCR chemokine receptor binding	3/28	0, 06	2, 6x10^-5^	MF
GO:0008009	chemokine activity	3/38	0, 08	6, 5x10^-5^	MF
GO:0042379	chemokine receptor binding	3/50	0, 1	1, 5x10^-4^	MF
CRSwNP-NP vs CRSwNP-NM					
GO:0035962	response to interleukin-13	3/4	0, 02	5, 7x10^-7^	BP
GO:0048245	eosinophil chemotaxis	4/14	0, 07	7x10^-7^	BP
GO:0072677	eosinophil migration	4/17	0, 09	1, 6x10^-6^	BP
GO:0071346	cellular response to interferon-gamma	6/92	0, 49	8, 9x10^-6^	BP
GO:0034341	response to interferon-gamma	6/108	0, 57	2, 2x10^-5^	BP
GO:0070374	positive regulation of ERK1 and ERK2 cascade	6/157	0, 83	1, 8x10^-4^	BP
GO:0048020	CCR chemokine receptor binding	4/28	0, 14	1, 2x10^-5^	MF
GO:0005125	cytokine activity	6/128	0, 63	3, 8x10^-5^	MF
GO:0008009	chemokine activity	4/38	0, 19	5x10^-5^	MF
CRSwNP-NM vs CS-NM					
GO:0001501	skeletal system development	3/406	0, 45	9, 2x10^-3^	BP
Downregulated					
CRSwNP-NP vs CS-NM					
GO:0001580	detection of chemical stimulus involved in sensory perception of bitter taste	4/12	0, 13	6, 1x10^-6^	BP
GO:0050913	sensory perception of bitter taste	4/13	0, 14	8, 8x10^-6^	BP
GO:0019731	antibacterial humoral response	5/27	0, 29	9, 3x10^-6^	BP
CRSwNP-NP vs CRSwNP-NM					
GO:0009593	detection of chemical stimulus	6/37	0, 51	1x10^-5^	BP
GO:0001580	detection of chemical stimulus involved in sensory perception of bitter taste	12/12	4	1, 6x10^-5^	BP
GO:0050913	sensory perception of bitter taste	4/13	0, 18	2, 3x10^-5^	BP
CRSwNP-NM vs CS-NM					
GO:0042363	fat-soluble vitamin catabolic process	3/8	0, 07	7x10^-5^	BP
GO:0019369	arachidonic acid metabolic process	5/40	0, 44	7, 3x10^-5^	BP
GO:0042573	retinoic acid metabolic process	4/21	0, 23	7, 3x10^-5^	BP
GO:0008401	retinoic acid 4-hydroxylase activity	4/6	0, 07	2, 6x10^-7^	MF

Significant n refers to the number of DEGs associated with the respective function, annotated n to the total number of genes associated with the respective function in the GO database, expected n to the number of the annotated genes that would appear by chance.

BP, biological process; CCR, chemokine receptor; CRSwNP-NM, paired nasal mucosa from patients with CRSwNP; CRSwNP-NP, nasal polyps from patients with CRSwNP; CS-NM, nasal mucosa from control subjects; DEG, differentially expressed gene; ERK pathway, extracellular-signal-regulated kinase pathway; GO, gene ontology; MF, molecular function.

### Molecular mechanisms in recurrent CRSwNP development predicted by KEGG and reactome pathway analysis

**Figure 4:**
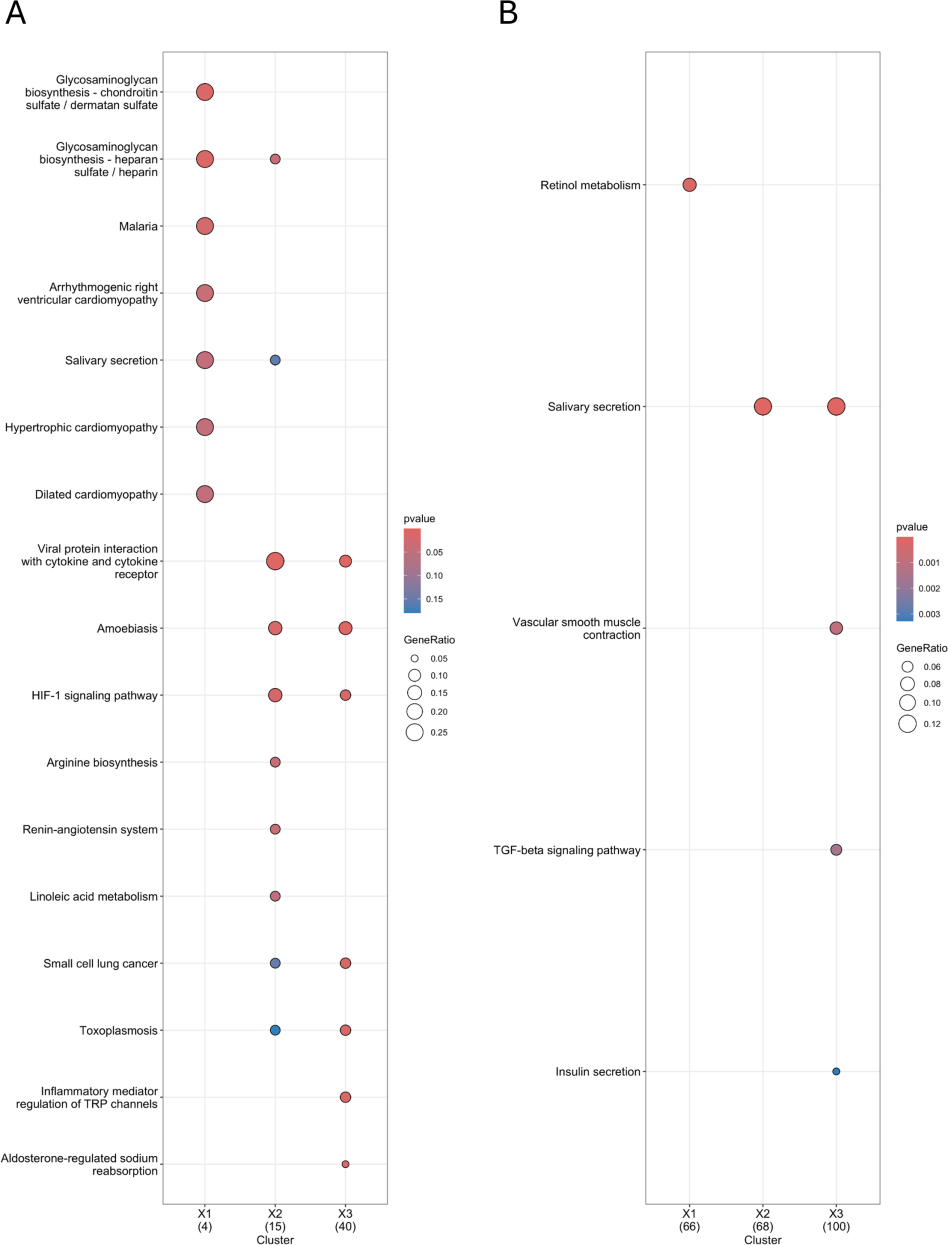
KEGG pathway analysis in patients with recurrent CRSwNP by comparing their self-controls and non-CRS nasal mucosa samples using cluster analysis **(A, B)** (colored) Dot plots show enriched KEGG pathways for upregulated DEGs **(A)** and downregulated **(B)** DEGs. Comparisons include X1: CRSwNP-NM and CS-NM, X2: CRSwNP-NP and CS-NM, and X3: CRSwNP-NP and CRSwNP-NM. Color scale: p-value calculated by hypergeometric test during overrepresentation analysis. Diameter of circles: proportional to the gene ratio (number of genes associated with the term in the differentially expressed gene set divided by the total number of differentially expressed genes in the respective comparison). CRSwNP-NM, nasal mucosa from patients with chronic rhinosinusitis with nasal polyps; CRSwNP-NP, nasal polyps from patients with chronic rhinosinusitis with nasal polyps; CS-NM, nasal mucosa from control subject; KEGG, Kyoto Encyclopedia of Genes and Genomes.

Compared to both nasal mucosa samples, KEGG pathway analysis of nasal polyp tissue revealed the most upregulated DEGs were primarily enriched in the viral protein interaction with cytokines and cytokine receptors, as well as the HIF-1 signaling pathway. Other notable enriched pathways in the comparison between CRSwNP-NP and CRSwNP-NM included inflammatory mediator regulation of transient receptor potential (TRP) channels and aldosterone-regulated sodium reabsorption. When comparing CRSwNP-NP to CS-NM, significant enrichment was observed in arginine biosynthesis, the renin-angiotensin system, and glycosaminoglycan (GAG) biosynthesis. The most downregulated DEGs in nasal polyp tissue, in both mucosal comparisons, were involved in salivary secretion and ATP-binding cassette (ABC) transporters. Comparing CRSwNP-NM with CS-NM, significant differences were observed, with upregulation in glycan biosynthesis and downregulation in retinol metabolism (**Figure 4**).

**Figure 5:**
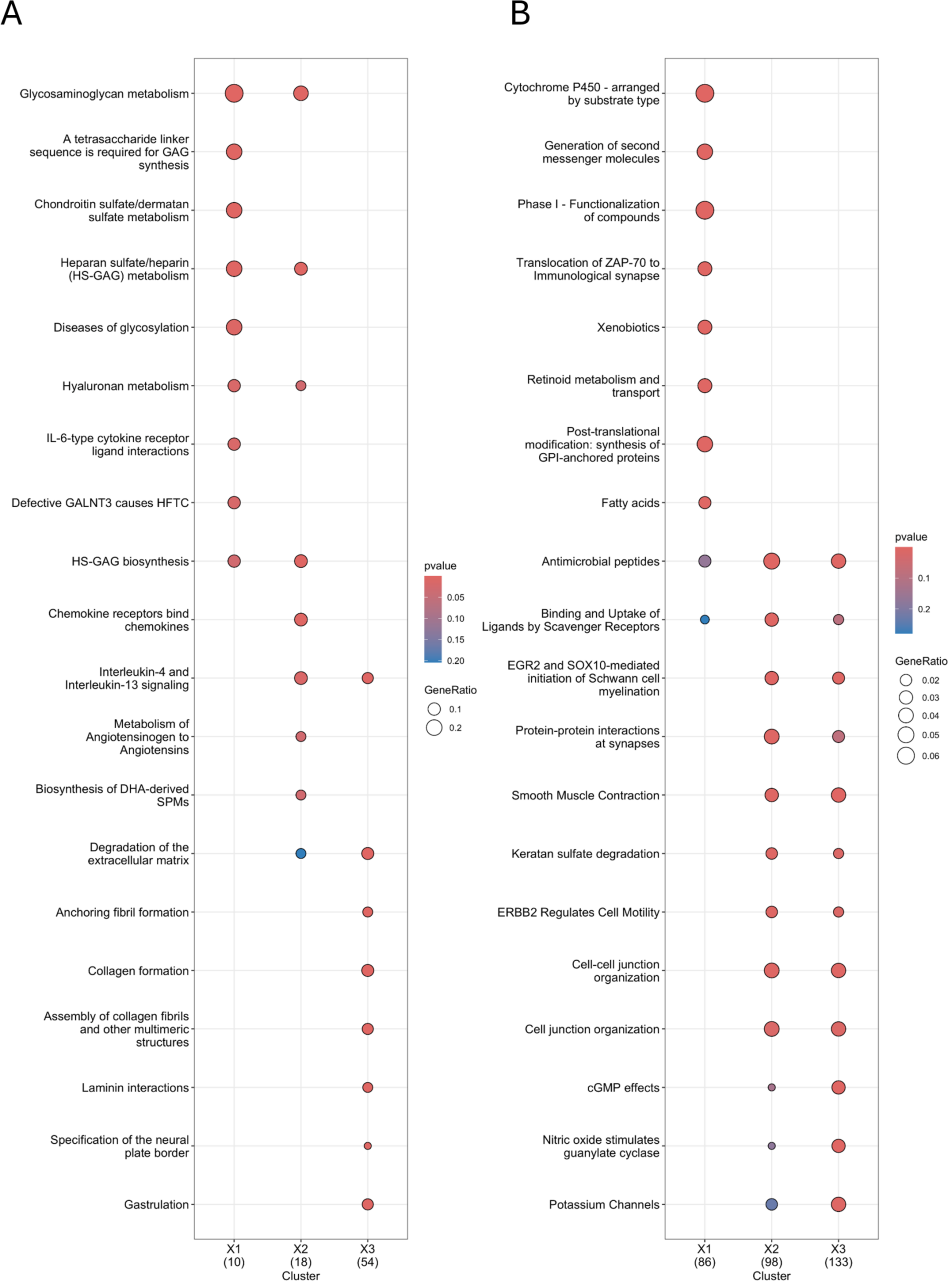
Reactome pathway analysis in patients with recurrent CRSwNP by comparing their self-controls and non-CRS nasal mucosa samples using cluster analysis **(A, B)** (colored) Dot plots represent enriched Reactome pathways for **(A)** upregulated DEGs and **(B)** downregulated DEGs. Comparisons include X1: CRSwNP-NM and CS-NM, X2: CRSwNP-NP and CS-NM, and X3: CRSwNP-NP and CRSwNP-NM. Color scale: p-value calculated by hypergeometric test during overrepresentation analysis. Diameter of circles: proportional to the gene ratio (number of genes associated with the term in the differentially expressed gene set divided by the total number of differentially expressed genes in the respective comparison). CRSwNP-NM, nasal mucosa from patients with chronic rhinosinusitis with nasal polyps; CRSwNP-NP, nasal polyps from patients with chronic rhinosinusitis with nasal polyps; CS-NM, nasal mucosa from control subject; DEG, differentially expressed gene.

Reactome pathway analysis revealed significantly upregulated DEGs in nasal polyp tissue in both comparisons, primarily related to signaling and ligand binding by G-protein–coupled receptors (GPCRs), whereas the most downregulated genes were associated with antiviral peptide production. Comparing CRSwNP-NP with CS-NM, the most upregulated DEGs were enriched also in GAG metabolism, heparan sulfate/heparin (HS-GAG) biosynthesis and metabolism. Interestingly, GAG and HS/GAG metabolism were also significantly upregulated in the CRSwNP-NM vs. CS-NM comparison. The downregulation of cytochrome P450 and xenobiotics was observed in the same comparison (**Figure 5**).

### Significantly enriched canonical pathways in recurrent CRSwNP development predicted by IPA

**Figure 6:**
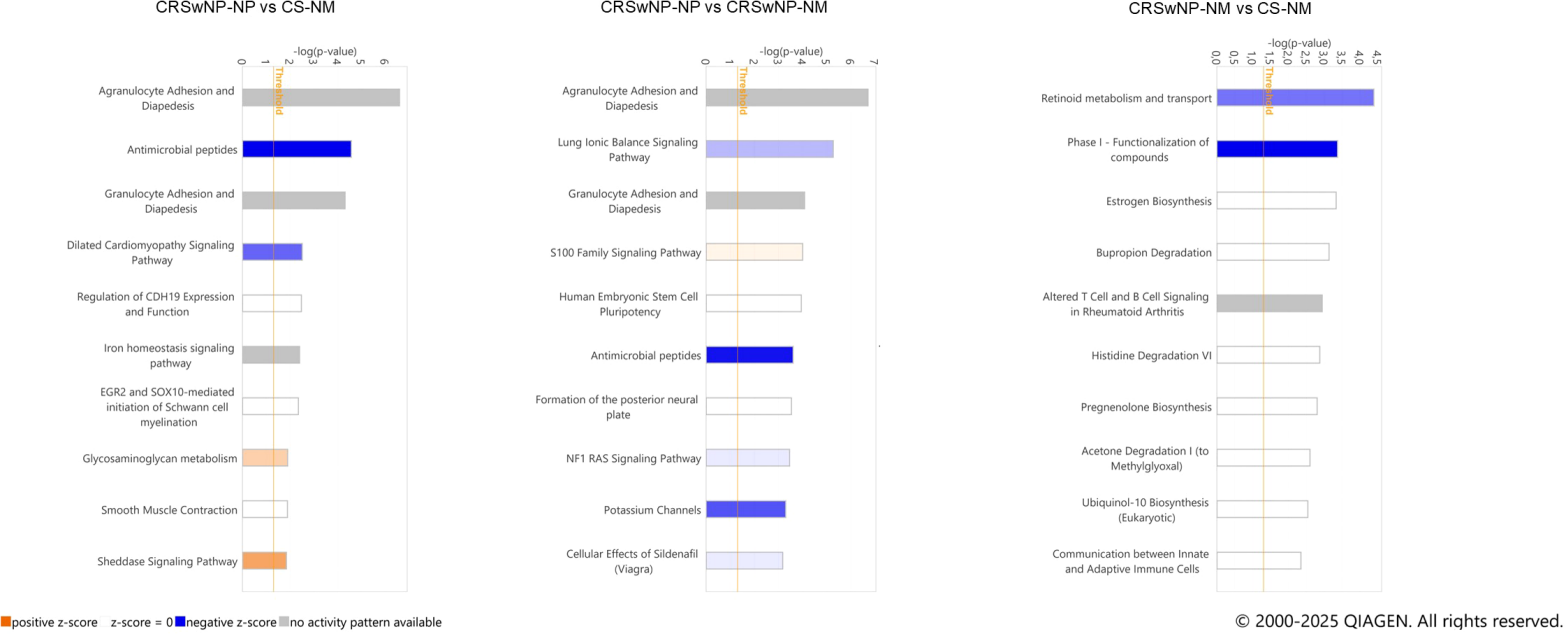
IPA revealed canonical pathways in patients with recurrent CRSwNP, determined through cluster analysis comparing self-control and non-CRS nasal mucosa samples (colored) Horizontal bars represent the top 10 canonical pathways ranked by –log(p-value) in each comparison. Color scale: a positive Z-score represents strongly activated (Z-score > 2) pathways, and a negative Z-score represents significantly inhibited (Z-score < −2) pathways. CRSwNP, chronic rhinosinusitis with nasal polyps; IPA, ingenuity pathway analysis.

Analysis using IPA revealed Agranulocyte Adhesion and Diapedesis, Granulocyte Adhesion and Diapedesis, and the Antimicrobial Peptides pathway as the most activated ones in CRSwNP-NP samples in both comparisons. The S100 Family pathway was the most upregulated in CRSwNP-NP compared with CRSwNP-NM. In addition, in the CRSwNP-NP vs. CS-NM comparison, glycosaminoglycan (GAG) metabolism and sheddase signaling pathways were among the most upregulated. Conversely, analysis of CRSwNP-NM vs. CS-NM samples showed, consistent with the Reactome results, that retinoid metabolism and transport were the most downregulated pathways (**Figure 6**).

### Identification of upstream regulators in recurrent CRSwNP nasal polyp samples using IPA

Among the relevant upstream regulators, we identified mirdametinib, tretinoin, adezmapimod, the synthetic aminoalkylindole cannabinoid receptor agonist WIN 55, 212-2, as well as naltrexone, doxycycline, and dexamethasone.

## Discussion

This is the first transcriptomic analysis of nasal polyps in patients with recurrent CRSwNP using double comparisons with self-control nasal mucosa and non-CRS samples employing complex bioinformatics tools across multiple databases in a White European population. Previous studies were limited to mostly Asian cohorts or European participants without self-controls, focused on CRSwNP vs. CRSsNP comparisons, and examined subpopulations of CRSwNP patients with comorbid asthma (7–12, 16, 23–28). This unbiased omics approach combined with bioinformatics is particularly useful to elucidate complex pathophysiological mechanisms and networks, as well as to identify molecular pathways for potential therapeutic interventions in various diseases. We used four synergistic databases—GO functional analysis, KEGG, Reactome and IPA enrichment analysis—to uncover biological processes, molecular functions, signaling pathways and interaction networks involved in CRSwNP pathogenesis.

The sequencing library type used in this study does not capture alternative splicing or isoform-specific expression. This is in accordance with the downstream analyses performed - differential expression testing and functional enrichment using the above-mentioned tools - are inherently gene-centric. Isoform-level resolution is difficult to interpret biologically in large-scale pathway analyses and was therefore outside the scope of the present work. Elucidating the precise nature of pathway interactions requires mechanistic follow-up studies, which we propose as future directions.

CLC was one of the most upregulated genes in nasal polyps in both comparisons representing a tissue-specific signature in agreement with earlier literature. It is a biomarker for eosinophilic inflammation in nasal secretions predicting CRSwNP recurrence (29). Cytokine and chemokine activity, along with CCR receptor binding, were also predominantly upregulated supported by previous studies (11, 16, 24). Upregulation of CCL13, CCL18 and CCL26 promote eosinophil degranulation, activate fibroblasts, induce the secretion of eosinophilotatic molecules, and recruit mast cells, NK cells, T cells and B cells, which are related to type 2 inflammation (30–33).

Transient receptor potential vanilloid type 1 (TRPV1) and type 4 (TRPV4) channels were upregulated in nasal polyp samples compared to nasal mucosa from the same patients. Both are non-selective cation channels (34) that play crucial roles in the release of pro-inflammatory sensory neuropeptides and cytokines (35). They are activated by a broad range of tissue irritants, leading to vasodilation and mucus secretion, thereby contributing to neurogenic inflammation (36, 37). TRPV1 channels induce thymic stromal lymphopoietin (TSLP) production in human nasal epithelial cell lines (38). TSLP is an epithelial-derived cytokine implicated in the initiation and maintenance of type 2 inflammatory pathways. Reducing TSLP synthesis by TRPV1 antagonists might be a potential therapeutic approach, but it has not yet been translated into clinical applications.

Statherin (STATH) was one of the most downregulated DEGs in nasal polyps supported by earlier data (24). STATH, along with LPO and DMBT1, reduce salivary secretion (24, 27). These genes encode proteins with Gram-negative and positive antimicrobial functions on mucosal surfaces (10). Dysregulation of the salivary secretion pathway can impair the innate immune response, increase the susceptibility to microbial colonization and persistent inflammation (27). MUC7 is the only DEG that shows significant differences across all comparisons, being downregulated in the polyp and upregulated in the normal nasal mucosa. It is a human salivary protein playing a role in the innate immune system (39, 40). MUC7 and its metal complexes exert potent anti-bacterial activities (41). MUC7 polymorphisms have previously been linked to bronchial asthma, with both protective (MUC7*5) and risk-associated VNTR variants described; however, these studies were limited to genetic variation and did not include transcriptomic evaluation (42, 43). The role of MUC7 in the pathogenesis of CRSwNP remains unclear. Previous studies have reported inconsistent findings, with some showing no detectable expression in normal nasal mucosa but increased expression in polyp tissue (44), while others failed to detect MUC7 in normal mucosa yet observed strong expression in nasal polyps (24). Our results support the previous one, the reduced MUC7 expression observed in nasal polyps may reflect impaired mucosal immune defense and further supports the relevance and complexity of compromised antimicrobial mechanisms in CRSwNP. PIP affects cell migration and adhesion (45), binding to CD4 receptors on lymphocytes and *Staphylococcus* bacteria (10). AZGP1 strongly correlates with CRS severity, particularly with VAS and SNOT22 scores (46). Furthermore, dexamethasone stimulates AZGP1 production, which may provide a novel explanation for the efficacy of glucocorticoid therapy in CRSwNP (47).

Epithelial-Mesenchymal Transition (EMT) refers to the condition in which epithelial cells acquire mesenchymal characteristics, lose cell-cell interactions and apicobasal polarity (48). This process is a key driver of nasal mucosal remodeling including several signaling pathways such as ERK and HIF-1α demonstrated by our results (49, 50). The ERK signaling pathway was one of the most upregulated pathways in the comparison between CRSwNP-NP and CS-NM. It can be activated by key cytokines involved in CRSwNP, such as IL-4, IL-13, IFN-γ, and transforming growth factor-β (TGF-β) (49, 50), leading to goblet cell hyperplasia and increased mucin production. Its activation results morphological changes and beating function of ciliary cells (51). Moreover, a positive correlation was observed between ERK expression and disease severity (52). Members of the sheddase signaling pathway are extracellular proteases that play a pivotal role in remodeling the extracellular matrix and initiate the shedding of transmembrane proteins at the plasma membrane. They regulate signalling pathways such as ERK1/2. To our knowledge, activation of this top-ranked signaling pathway has not previously been reported in CRSwNP. The observed overactivation of this pathway further supports the involvement of EMT in CRSwNP pathogenesis, which is an interesting target for future therapies. The S100 family signaling pathway was also among the top-ranked canonical pathways in the comparison between CRSwNP-NP and CRSwNP-NM, supported by previous findings (53). S100 family proteins are damage-associated molecular patterns (DAMPs) with potent proinflammatory activities. These proteins can also activate the ERK pathway, thereby further enhancing EMT (54). HIF-1 signaling was among the most prominently upregulated pathways in nasal polyps in both comparisons, consistent with previous reports (10, 25, 28, 55, 56). Activation of the HIF-1 pathway is linked to hypoxia-induced EMT but can also be triggered by IFN-γ under normoxic conditions (57). Hypoxia represents a key driver of inflammation through multiple mechanisms: polyp mass can compress local vessels, nasal dysbiosis can aggravate tissue hypoxia and perpetuate inflammation, or conversely, pathogen invasion itself can induce hypoxia (58, 59). The hypoxic environment influences the proliferation and differentiation of mast cells, macrophages, eosinophils, basophils, and epithelial cells, leading to impaired mucociliary clearance and barrier function. Consequently, pathogen invasion and epithelial immune activation are enhanced, triggering pro-inflammatory cytokine release. The HIF-1α subunit, the central transcriptional regulator under hypoxia, upregulates glycolytic enzymes (60), increases vascular permeability, and promotes myofibroblast differentiation, extracellular matrix production, and EMT (55).

In the human sinonasal epithelium, nitric oxide (NO) is synthesized from oxygen and L-arginine by inducible nitric oxide synthase (iNOS) (61), which has antimicrobial properties and plays a role in immune responses and inflammation (61). Several studies have reported increased iNOS activity and elevated NO levels in response to inflammatory stimuli (61). Consistent with other transcriptomic studies (10, 62), we found significant overactivation of arginine synthesis, which is essential for NO production, in nasal polyps.

Ferroptosis is an iron-dependent, oxidatively regulated form of cell death, where toxic accumulation of lipid-reactive oxygen species and unsaturated fatty acids trigger lipid peroxidation in the cell membrane (62). The process is involved in inflammatory responses, among others, but its role in the pathogenesis of CRS has not yet been fully elucidated. KEGG pathway analysis revealed increased activity of the ALOX15 gene underlying ferroptosis upregulation, consistent with the findings of others (53).

The role of RAAS in CRS pathogenesis is well established, through its activation leading to periostin upregulation (63). Some retrospective studies have reported that RAAS inhibitors (angiotensin-converting enzyme inhibitors and angiotensin receptor blockers) can improve quality of life, reduce polyp size and postoperative recurrence, stabilize nasal symptoms (63, 64). However, the transcriptomic mechanisms underlying these effects remain incompletely understood. Our results revealed significant upregulation of RAAS and aldosterone-regulated sodium reabsorption pathways in nasal polyps, supporting the role of RAAS overactivation in disease pathogenesis.

GAGs are a class of innate immune system modulators and key components of the ECM. Hyaluronic acid (HA), a non-sulphated GAG, exists in high-molecular-weight form, which is physiologically available and has anti-inflammatory effects, and its short-fragment, which exhibits pro-inflammatory effects (65). Sulphated GAGs, such as heparan- and chondroitin-sulfate, inhibit key inflammatory mediators, promote mucosal surface repair, and enhance mucociliary clearance (66). Our enrichment analysis — including both KEGG and Reactome — revealed upregulation of sulphated GAG biosynthesis and metabolism in both CRSwNP patient samples compared to control mucosa. In addition, we observed a significant upregulation of heparin and heparan sulfate biosynthesis and metabolism in the comparison between polyp tissue and non-CRS nasal mucosa. While the role of GAGs is well established in the pathophysiology of several inflammatory pulmonary diseases. A study published in 2005 analyzed the glycosaminoglycan composition of nasal polyp tissues using column chromatography and reported a marked increase in hyaluronic acid levels (67); however, no mechanistic explanation or causal relationship was identified, and to the best of our knowledge, beyond these reports, the role of glycosaminoglycans in the pathogenesis of chronic rhinosinusitis has not been explored in greater depth, thereby limiting direct comparison with our findings. The upregulated GAG biosynthesis observed in nasal polyps may be explained by the overproduction of low–molecular-weight hyaluronic acid (HA), whereas the upregulation detected in the intact nasal mucosa of CRSwNP patients may represent a susceptibility-related alteration contributing to disease development. Future studies integrating molecular weight–specific HA profiling and functional analyses will be important to clarify the role of distinct HA fractions in disease initiation, tissue remodeling, and recurrence risk.

ABC transporters constitute one of the largest and most ancient transporter superfamilies, responsible for the ATP-dependent translocation of various substrates across cellular membranes (68). Despite their broad importance, their role in CRS remains poorly understood, with contradictory findings reported in the literature. The ABC transporter subfamily C member 1 (ABCC1) encodes multidrug resistance protein (MRP), which exists in nine isoforms (MRP1–9). MRPs have been detected in the olfactory epithelium and nasal polyps (69) and have been associated with both pro- and anti-inflammatory functions (70). In our study, ABC transporters (ABCC3, 8–10) were significantly downregulated in nasal polyps compared to both control groups, supporting previous observations that HIF-1 overexpression can lead to reduced ABCG2 expression (71).

Altered transcriptomic signatures detected in macroscopically normal mucosa from CRSwNP patients may reflect systemic susceptibility factors contributing to disease development. In addition to the aforementioned changes in GAG metabolism, the most notable alteration was the downregulation of retinol metabolism in the nasal mucosa of CRSwNP patients compared to that of control subjects. Previous studies have reported reduced levels of vitamin A and retinoic acid (RA) exclusively in nasal polyp tissue (72). The reduced retinoid metabolism observed in CRSwNP-NM samples may be explained by pronounced fibrin deposition. Excessive fibrin accumulation—one of the hallmarks of CRSwNP pathogenesis—has been associated with lower levels of epithelial tissue plasminogen activator (tPA), as its activation is retinoid dependent (72). Consequently, decreased retinoid levels may reduce tPA activity, thereby promoting fibrin deposition and contributing to polyp formation. This alteration may be associated with an increased susceptibility to disease. No significant difference was observed between CRSwNP polyp tissue and mucosal samples.

*In vivo* and *in vitro* experiments have confirmed that vitamin A derivatives attenuate type 2 inflammatory responses, increase the anti-inflammatory cytokine IL-10, and modulate extracellular matrix production and tissue remodeling (73, 74). In a randomized, open-label clinical trial, topical tretinoin was administered to patients with CRSwNP based on its known immunomodulatory effects, including suppression of pro-inflammatory mediators, without accompanying transcriptomic evaluation (74). Treatment was associated with improved olfactory function and reduced microscopic tissue oedema. To date, no studies have directly demonstrated alterations in retinoid metabolism or impairments in retinoid transport in patients with CRSwNP as a systemic susceptibility factor. In this context, our transcriptomic findings provide novel evidence suggesting dysregulation of retinoid metabolic and transport pathways in CRSwNP. Accordingly, future prospective clinical studies or interventional trials are warranted to determine whether modulation of retinol metabolism influences disease recurrence or treatment response.

Among the most prominent upstream regulators identified in nasal polyp tissue, dexamethasone - one of the cornerstone therapies in the management of CRSwNP - and doxycycline - known modulator of type 2 inflammation - were detected, providing internal validation of our findings. In addition, two MAPK inhibitors were identified, which are consistent with our transcriptomic results and suggest that preclinical evaluation of these agents may be warranted, as such studies have not yet been reported in the context of CRSwNP to our knowledge. Furthermore, the identification of tretinoin as a potent upstream regulator further supports the relevance of our results. The analysis also identified unexpected upstream regulators, such as naltrexone—primarily used in the treatment of alcohol and opioid dependence—as well as a synthetic aminoalkylindole cannabinoid receptor agonist, WIN 55, 212-2. WIN 55, 212–2 has been reported to inhibit p38 MAPK activation in human astrocytes and in experimental rodent models (75, 76). These findings point to previously unexplored mechanisms that may warrant further investigation, which can provide new targeted therapeutic approaches.

### Limitations

This study has several limitations, including a relatively small, single-center cohort consisting only of White European patients, which may limit generalizability. The relatively small cohort size can be partly explained by the substantial overlap between the study period and the COVID-19 pandemic, as well as by the increasingly widespread use of biological therapies. Control samples were obtained from different sinonasal sites, potentially introducing biological heterogeneity. Regarding comorbidities associated with CRSwNP, the prevalence of bronchial asthma was comparable to that reported for both patient and control populations (77). In contrast, the prevalence of allergic rhinitis was lower than typically reported (11–16%), with an observed prevalence of 6.7% in our cohort (78). Functional validation was not performed, as no well-established cell line or animal model currently exists that reliably captures the complexity of CRSwNP, particularly in its recurrent form, which was the focus of the present study. As the analysis is based on transcriptomic profiling, the identified genes and pathways should be regarded as hypothesis-generating and require further functional validation. In addition, the cross-sectional study design does not allow conclusions regarding causality or disease dynamics, underscoring the need for longitudinal studies to clarify the role of glycosaminoglycan and retinoid pathways in CRSwNP pathogenesis.

## Conclusions

By employing multiple, synergistic bioinformatic approaches with self-control and non-CRS nasal mucosa comparisons, this study provides an integrative and comprehensive analysis of gene expression profiles in nasal polyps, enabling the identification of key pathophysiological pathways underlying CRSwNP. Our findings reveal the involvement of multiple, interconnected mechanisms, including type 2 inflammation, tissue remodeling, impaired antibacterial defense, hypoxia, TRPV channel upregulation and dysregulated RAAS signaling. Based on current evidence, the latter two modifications appear to have promising therapeutic potentials. TRPV channel antagonists may represent promising therapeutic options through the reduction of TSLP. Our results support the need for further, in-depth investigation of the therapeutic efficacy of RAAS inhibitors. Moreover, as a principal novelty of our study lies in the observed upregulation of GAG biosynthesis and metabolism, as well as the altered expression of retinol metabolism in the histologically normal nasal mucosa of patients with CRSwNP, suggesting systemic susceptibility factors, emphasizing that CRSwNP is shaped not only by local tissue alterations but also by intrinsic patient-specific vulnerabilities.

These findings not only clarify underexplored molecular mechanisms but also define actionable targets for innovative therapeutic strategies aimed at modulating both local remodeling and systemic vulnerabilities in the future.

## Data Availability

The datasets presented in this study can be found in online repositories. The names of the repository/repositories and accession number(s) can be found below: PRJEB88945 (ENA; https://www.ebi.ac.uk/ena/browser/view/PRJEB88945).

## References

[1] Fokkens WJ Lund VJ Hopkins C Hellings PW Kern R Reitsma S . European position paper on rhinosinusitis and nasal polyps 2020. Rhinology. (2020) 58:1–464. doi: 10.4193/Rhin20.600, PMID: 32077450

[2] Staudacher AG Peters AT Kato A Stevens WW . Use of endotypes, phenotypes, and inflammatory markers to guide treatment decisions in chronic rhinosinusitis. Ann Allergy Asthma Immunol. (2020) 124:318–25. doi: 10.1016/j.anai.2020.01.013, PMID: 32007571 PMC7192133

[3] Grayson JW Hopkins C Mori E Senior B Harvey RJ . Contemporary classification of chronic rhinosinusitis beyond polyps vs. No polyps: A review. JAMA Otolaryngol Head Neck Surg. (2020) 146:831–8. doi: 10.1001/jamaoto.2020.1453, PMID: 32644117

[4] Bassiouni A Naidoo Y Wormald PJ . The inflammatory load hypothesis in refractory chronic rhinosinusitis. Laryngoscope. (2012) 122:460–6. doi: 10.1002/lary.22461, PMID: 22252862

[5] Wang Z Gerstein M Snyder M . RNA-Seq: a revolutionary tool for transcriptomics. Nat Rev Genet. (2009) 10:57–63. doi: 10.1038/nrg2484, PMID: 19015660 PMC2949280

[6] Stark R Grzelak M Hadfield J . RNA sequencing: the teenage years. Nat Rev Genet. (2019) 20:631–56. doi: 10.1038/s41576-019-0150-2, PMID: 31341269

[7] Wang W Gao Z Wang H Li T He W Li P . Transcriptome analysis reveals distinct gene expression profiles in eosinophilic and noneosinophilic chronic rhinosinusitis with nasal polyps. Sci Rep. (2016) 6:26604. doi: 10.1038/srep26604, PMID: 27216292 PMC4877582

[8] Bassiouni A Ou J Schreiber A Geoghegan J Tsykin A Vreugde S . The global transcriptomic signature in sinonasal tissues reveals roles for tissue type and chronic rhinosinusitis disease phenotype. Rhinology. (2020) 58:273–83. doi: 10.4193/Rhin19.403, PMID: 32147672

[9] Soklič TK Rijavec M Silar M Koren A Kern I Hocevar-Boltezar I . Transcription factors gene expression in chronic rhinosinusitis with and without nasal polyps. Radiol Oncol. (2019) 53:323–30. doi: 10.2478/raon-2019-0029, PMID: 31326962 PMC6765166

[10] Urbančič J Soklič TK Demšar Luzar A Hočevar Boltežar I Korošec P Rijavec M . Transcriptomic differentiation of phenotypes in chronic rhinosinusitis and its implications for understanding the underlying mechanisms. Int J Mol Sci. (2023) 24:5541. doi: 10.3390/ijms24065541, PMID: 36982612 PMC10051401

[11] Hao Y Zhao Y Wang P Du K Li Y Yang Z . Transcriptomic signatures and functional network analysis of chronic rhinosinusitis with nasal polyps. Front Genet. (2021) 12:609754. doi: 10.3389/fgene.2021.609754, PMID: 33603773 PMC7884819

[12] Peng Y Zi XX Tian TF Lee B Lum J Tang SA . Whole-transcriptome sequencing reveals heightened inflammation and defective host defence responses in chronic rhinosinusitis with nasal polyps. Eur Respir J. (2019) 54:1900732. doi: 10.1183/13993003.00732-2019, PMID: 31439685

[13] Jeong SS Chen T Nguyen SA Edwards TS Schlosser RJ . Correlation of polyp grading scales with patient symptom scores and olfaction in chronic rhinosinusitis: a systematic review and meta-analysis. Rhinology. (2022) 58:322–34. doi: 10.4193/Rhin22.011, PMID: 36191585

[14] Lund VJ Kennedy DW . Staging for rhinosinusitis. Otolaryngol Head Neck Surg. (1997) 117:S35–40. doi: 10.1016/S0194-5998(97)70005-6, PMID: 9334786

[15] Washington University in St. Louis . Sino-Nasal Outcome Test (SNOT-22) – Available translations. Available online at: https://sinonasaltest.wustl.edu (Accessed March 15, 2026).

[16] Wang K Deng J Yang M Chen Y Chen F Gao WX . Concordant systemic and local eosinophilia relates to poorer disease control in patients with nasal polyps. World Allergy Organ J. (2019) 12:100052. doi: 10.1016/j.waojou.2019.100052, PMID: 31452832 PMC6704051

[17] Busnell B Rood J Singer E . BBMerge – Accurate paired shotgun read merging via overlap. PloS One. (2017) 12:e0185056. doi: 10.1371/journal.pone.0185056, PMID: 29073143 PMC5657622

[18] Anders S Pyl PT Huber W . HTSeq--a Python framework to work with high-throughput sequencing data. Bioinform. (2015) 31:166–9. doi: 10.1093/bioinformatics/btu638, PMID: 25260700 PMC4287950

[19] Ritchie ME Phipson B Wu D Hu Y Law CW Smyth GK . limma powers differential expression analyses for RNA-sequencing and microarray studies. Nucleic Acids Res. (2015) 20:e47. doi: 10.1093/nar/gkv007, PMID: 25605792 PMC4402510

[20] Dobin A Davis CA Schlesinger F Drenkow J Zaleski C Jha S . STAR: ultrafast universal RNA-seq aligner. Bioinformatics. (2013) 29:15–21. doi: 10.1093/bioinformatics/bts635, PMID: 23104886 PMC3530905

[21] Toma S Hopkins C . Stratification of SNOT-22 scores into mild, moderate or severe and relationship with other subjective instruments. Rhinology. (2016) 54:129–33. doi: 10.4193/Rhino15.072, PMID: 27017484

[22] Brar T McCabe C Miglani A Marino M Lal D . Tissue eosinophilia is superior to an analysis by polyp status for the chronic rhinosinusitis transcriptome: an RNA study. Laryngoscope. (2023) 133:2480–9. doi: 10.1002/lary.30544, PMID: 36594502

[23] Wang M Tang S Yang X Xie X Luo Y He S . Identification of key genes and pathways in chronic rhinosinusitis with nasal polyps and asthma comorbidity using bioinformatics approaches. Front Immunol. (2022) 13:941547.23. doi: 10.3389/fimmu.2022.941547, PMID: 36059464 PMC9428751

[24] Ishino T Takeno S Takemoto K Yamato K Oda T Nishida M . Distinct gene set enrichment profiles in eosinophilic and non-eosinophilic chronic rhinosinusitis with nasal polyps by bulk RNA barcoding and sequencing. Int J Mol Sci. (2022) 23:5653. doi: 10.3390/ijms23105653, PMID: 35628459 PMC9146754

[25] Chen G Hao H Wang LE . Bioinformatics analysis and verification of key candidate genes influencing the pathogenesis of chronic rhinosinusitis with nasal polyps. Am J Transl Res. (2023) 15:710–28. PMC10006810 36915741

[26] Yao Y Xie S Wang F . Identification of key genes and pathways in chronic rhinosinusitis with nasal polyps using bioinformatics analysis. Am J Otolaryngol. (2019) 40:191–6. doi: 10.1016/j.amjoto.2018.12.002, PMID: 30661889

[27] Zhou X Zhen X Liu Y Cui Z Yue Z Xu A . Identification of key modules, hub genes, and noncoding RNAs in chronic rhinosinusitis with nasal polyps by weighted gene coexpression network analysis. BioMed Res Int. (2020) 2020:6140728. doi: 10.1155/2020/6140728, PMID: 32047813 PMC7003281

[28] Wu D Yan B Wang Y Zhang L Wang C . Predictive significance of charcot-leyden crystal protein in nasal secretions in recurrent chronic rhinosinusitis with nasal polyps. Int Arch Allergy Immunol. (2021) 182:65–75. doi: 10.1159/000510120, PMID: 32927462

[29] Nakayama T Lee IT Le W Tsunemi Y Borchard NA Zarabanda D . Inflammatory molecular endotypes of nasal polyps derived from White and Japanese populations. J Allergy Clin Immunol. (2022) 149:1296–1308.e6. doi: 10.1016/j.jaci.2021.11.017, PMID: 34863854

[30] Li L Dai F Wang L Sun Y Mei L Ran Y . CCL13 and human diseases. Front Immunol. (2023) 14:1176639. doi: 10.3389/fimmu.2023.1176639, PMID: 37153575 PMC10154514

[31] Peterson S Poposki JA Nagarkar DR Chustz RT Peters AT Suh LA . Increased expression of CC chemokine ligand 18 in patients with chronic rhinosinusitis with nasal polyps. J Allergy Clin Immunol. (2012) 129:119–127.e1-9. doi: 10.1016/j.jaci.2011.08.021, PMID: 21943944 PMC3246095

[32] El-Shazly AE Doloriert HC Bisig B Lefebvre PP Delvenne P Jacobs N . Novel cooperation between CX3CL1 and CCL26 inducing NK cell chemotaxis via CX3CR1: a possible mechanism for NK cell infiltration of the allergic nasal tissue. Clin Exp Allergy. (2012) 43:322–31. doi: 10.1111/cea.12022, PMID: 23414540

[33] Silverman HA Chen A Kravatz NL Chavan SS Chang EH . Involvement of neural transient receptor potential channels in peripheral inflammation. Front Immunol. (2020) 11:590261. doi: 10.3389/fimmu.2020.590261, PMID: 33193423 PMC7645044

[34] Backaert W Steelant B Hellings PW Talavera K Van Gerven L . A TRiP through the roles of transient receptor potential cation channels in type 2 upper airway inflammation. Curr Allergy Asthma Rep. (2021) 21:20. doi: 10.1007/s11882-020-00981-x, PMID: 33738577 PMC7973410

[35] Bhargave G Woodworth BA Xiong G Wolfe SG Antunes MB Cohen NA . Transient receptor potential vanilloid type 4 channel expression in chronic rhinosinusitis. Am J Rhinol. (2008) 22:7–12. doi: 10.2500/ajr.2008.22.3125, PMID: 18284852

[36] Lawhorn BG Brnardic EJ Behm DJ . Recent advances in TRPV4 agonists and antagonists. Bioorg Med Chem Lett. (2020) 30:127022. doi: 10.1016/j.bmcl.2020.127022, PMID: 32063431

[37] Li J Wang F Meng C Zhu D . Role of TRPV1 and TRPA1 in TSLP production in nasal epithelial cells. Int Immunopharmacol. (2024) 131:111916. doi: 10.1016/j.intimp.2024.111916, PMID: 38522138

[38] Ali MJ Paulsen F . Prolactin and Prolactin-inducible protein (PIP) in the pathogenesis of primary acquired nasolacrimal duct obstruction (PANDO). Med Hypotheses. (2019) 125:137–8. doi: 10.1016/j.mehy.2019.02.051, PMID: 30902142

[39] Tsai YJ Hsu YT Ma MC Wu CK Luo SD Wu WB . Transcriptomic analysis of genes associated with oxidative stress in chronic rhinosinusitis patients with nasal polyps: identifying novel genes involved in nasal polyposis. Antioxidants. (2022) 11:1899. doi: 10.3390/antiox11101899, PMID: 36290622 PMC9598890

[40] Janicka-Kłos A Czapor-Irzabek H Janek T . The potential antimicrobial action of human mucin 7 15-mer peptide and its metal complexes. Int J Mol Sci. (2022) 23:418. doi: 10.3390/ijms23010418, PMID: 35008844 PMC8745124

[41] Kirkbride H Bolscher J Nazmi K Vinall LE Nash MW Moss FM . Genetic polymorphism of MUC7: Allele frequencies and association with asthma. Eur J Hum Genet. (2001) 9:347–54. doi: 10.1038/sj.ejhg.5200642, PMID: 11378823

[42] Saad EA Elsaid AM Shoaib RMS Megahed KF Elsharawy AN . MUC7 VNTR polymorphism and association with bronchial asthma in Egyptian children. Sci Rep. (2022) 12:18910. doi: 10.1038/s41598-022-21631-4 36344553 PMC9640678

[43] Ali MS Wilson JA Bennett M Pearson JP . Mucin gene expression in nasal polyps. Acta Otolaryngol. (2005) 125:618–24. doi: 10.1080/00016480510027538, PMID: 16076710

[44] Naderi A Vanneste M . Prolactin-induced protein is required for cell cycle progression in breast cancer. Neoplasia. (2014) 16:329–42. doi: 10.1016/j.neo.2014.04.001, PMID: 24862759 PMC4094838

[45] Jin P Zhang Q Zang Y Zhao L Zhang H Yu K . Down regulation of EGF and AZGP1 were associated with clinical characteristics in chronic rhinosinusitis with nasal polyps: an observation study. J Inflammation Res. (2023) 16:4885–98. doi: 10.2147/JIR.S428238 PMC10619462 37920240

[46] Bing C Bao Y Jenkins J Sanders P Manieri M Cinti S . Zinc-alpha2-glycoprotein, a lipid mobilizing factor, is expressed in adipocytes and is up-regulated in mice with cancer cachexia. Proc Natl Acad Sci USA. (2004) 101:2500–5. doi: 10.1073/pnas.0308647100, PMID: 14983038 PMC356979

[47] Acloque H Adams HS Fishwick K Bronner-Fraser M Nieto MA . Epithelial-mesenchymal transitions: the importance of changing cell state in development and disease. J Clin Invest. (2009) 119:1438–49. doi: 10.1172/JCI38019, PMID: 19487820 PMC2689100

[48] Jing Y Wang M Wang C Zhang L . Epithelial cell dysfunction in chronic rhinosinusitis: the epithelial–mesenchymal transition. Expert Rev Clin Immun. (2023) 19:959–68. doi: 10.1080/1744666X.2023.2232113, PMID: 37386882

[49] Niu Z Sha J Zhu D Meng C . Investigation and characterization of the RAS/RAF/MEK/ERK pathway and other signaling pathways in chronic sinusitis with nasal polyps. Int Arch Allergy Immunol. (2025) 186:252–63. doi: 10.1159/000541041, PMID: 39353408

[50] Ma Y Tian P Zhong H Wu F Zhang Q Liu X . WDPCP modulates cilia beating through the MAPK/ERK pathway in chronic rhinosinusitis with nasal polyps. Front Cell Dev Bio. (2021) 8:630340. doi: 10.3389/fcell.2020.630340, PMID: 33598458 PMC7882705

[51] Xu M Chen Z Jiang Q Tang H . Role of CD23 activated ERK signaling pathway in the pathogenesis of eosinophilic chronic sinusitis with nasal polyps. Altern Ther Health Med. (2023) 29:638–43. 37678868

[52] Zou S Huang Z Wu J . Predictive value of S100A4 in eosinophilic chronic rhinosinusitis with nasal polyps. Front Surg. (2022) 9:989489. doi: 10.3389/fsurg.2022.989489, PMID: 36386522 PMC9663474

[53] Ahn SH Oh JT Kim DH Lee EJ Rha MS Cho HJ . S100A9 induces tissue remodeling of human nasal epithelium in chronic rhinosinusitis with nasal polyp. Int Forum Allergy Rhinol. (2025) 15:135–48. doi: 10.1002/alr.23460, PMID: 39367796 PMC11785152

[54] Xia Y Wang H Yin J . The role of epithelial-mesenchymal transition in chronic rhinosinusitis. Int Arch Allergy Immunol. (2022) 183:1029–39. doi: 10.1159/000524950, PMID: 35738243

[55] Jo MS Yang HW Park JH Shin JM Park IH . Glycolytic reprogramming is involved in tissue remodeling on chronic rhinosinusitis. PloS One. (2023) 18:e0281640. doi: 10.1371/journal.pone.0281640, PMID: 36795696 PMC9934430

[56] Chien CY Tai CF Ho KY Kuo WR Chai CY Hsu YC . Expression of hypoxia-inducible factor 1alpha in the nasal polyps by real-time RT-PCR and immunohistochemistry. Otolaryngol Head Neck Surg. (2008) 139:206–10. doi: 10.1016/j.otohns.2008.04.022, PMID: 18656716

[57] Xu W Ghosh S Comhair SA Asosingh K Janocha AJ Mavrakis DA . Increased mitochondrial arginine metabolism supports bioenergetics in asthma. J Clin Invest. (2016) 126:2465–81. doi: 10.1172/JCI82925, PMID: 27214549 PMC4922712

[58] Zhong B Seah JJ Liu F Ba L Du J Wang DY . The role of hypoxia in the pathophysiology of chronic rhinosinusitis. Allergy. (2022) 77:3217–32. doi: 10.1111/all.15384, PMID: 35603933

[59] Kierans SJ Taylor CT . Regulation of glycolysis by the hypoxia-inducible factor (HIF): implications for cellular physiology. J Physiol. (2021) 599:23–37. doi: 10.1113/JP280572, PMID: 33006160

[60] Zhu M Gao X Hu X Zhou H Liu J . The roles of nasal nitric oxide in diagnosis and endotypes of chronic rhinosinusitis with nasal polyps. J Otolaryngol - Head Neck Surg. (2020) 49:68. doi: 10.1186/s40463-020-00465-y, PMID: 32962755 PMC7507626

[61] Huang GJ Liu HB . Identification and validation of ferroptosis-related genes for chronic rhinosinusitis with nasal polyps. Eur Arch Oto-Rhino-Laryngol. (2022) 280:1501–8. doi: 10.1007/s00405-022-07696-x, PMID: 36255469

[62] Brook CD Maxfield AZ Stankovic K Metson RB . The impact of angiotensin-modulating antihypertensives on time interval to revision surgery for nasal polyps. Otolaryngol Head Neck Surg. (2016) 155:1046–52. doi: 10.1177/0194599816663924, PMID: 27554516

[63] Maza-Solano J Palma-Martínez C Martín-Jiménez D Sánchez-Gómez S Moreno-Luna R Calvo-Henriquez C . Effect of antihypertensive treatment on the quality of life of patients with chronic rhinosinusitis with nasal polyps. Acta Otorrinolaringol Esp. (English Edition). (2024) 75:155–61. doi: 10.1016/j.otoeng.2024.01.003, PMID: 38220051

[64] Garantziotis S Brezina M Castelnuovo P Drago L . The role of hyaluronan in the pathobiology and treatment of respiratory disease. Am J Physiol Lung Cell Mol Physiol. (2016) 310:L785–95. doi: 10.1152/ajplung.00168.2015, PMID: 26747781 PMC4867348

[65] Alt JA Lee WY Davis BM Savage JR Kennedy TP Prestwichet GD . A synthetic glycosaminoglycan reduces sinonasal inflammation in a murine model of chronic rhinosinusitis. PloS One. (2018) 13:e0204709. doi: 10.1371/journal.pone.0204709, PMID: 30252910 PMC6155557

[66] Pawłowska-Góral K Gierek T Majzel K Wardas P Wardas M . Glycosoaminoglycans in nasal polyps. Acta Oto-Laryngologica. (2005) 125:177–9. doi: 10.1080/00016480410017116, PMID: 15880949

[67] Higgins CF . ABC transporters: from microorganisms to man. Annu Rev Cell Biol. (1992) 8:67–113. doi: 10.1146/annurev.cb.08.110192.000435, PMID: 1282354

[68] Inuma T Yonekura S Hirahara K Kurita J Yoneda R Arai T . Differences in the expression of multidrug resistance proteins in chronic rhinosinusitis according to endotype. Allergol Int. (2023) 72:564–72. doi: 10.1016/j.alit.2023.03.008, PMID: 37147165

[69] Grigoreva TA Sagaidak AV Novikova DS Tribulovich VG . Implication of ABC transporters in non-proliferative diseases. Euro J Pharmacol. (2022) 935:175327. doi: 10.1016/j.ejphar.2022.175327, PMID: 36265610

[70] Francois LN Gorczyca L Du J Bircsak KM Yen E Zhou Y . Down-regulation of the placental BCRP/ABCG2 transporter in response to hypoxia signaling. Placenta. (2017) 51:57–63. doi: 10.1016/j.placenta.2017.01.125, PMID: 28292469 PMC5354084

[71] Sakashita M Takabayashi T Imoto Y Homma T Yoshida K Hara T . Retinoic acid promotes fibrinolysis and may regulate polyp formation. J Allergy Clin Immunol. (2022) 150:1114–24. doi: 10.1016/j.jaci.2022.05.021, PMID: 35728655 PMC11152199

[72] Lee HJ Kim DK . Retinoic acid treatment mitigates PM2.5-induced type 2 inflammation: insights into modulation of innate immune responses. Int J Mol Sci. (2024) 25:3856. doi: 10.3390/ijms25073856, PMID: 38612663 PMC11011870

[73] Kim SJ Park JH Lee SA Lee JG Shin JM Lee HM . All-trans retinoic acid regulates TGF-β1-induced extracellular matrix production via p38, JNK, and NF-κB-signaling pathways in nasal polyp-derived fibroblasts. Int Forum Allergy Rhinol. (2020) 10:636–45. doi: 10.1002/alr.22525, PMID: 32104972

[74] Antonio MA Marson FAL Toro MDC Sampaio MH Barreto IS Valera FCP . Topical tretinoin in chronic rhinosinusitis with nasal polyps: a randomized clinical trial. Int Forum Allergy Rhinol. (2021) 11:1187–96. doi: 10.1002/alr.22778, PMID: 33583149

[75] Sheng WS Hu S Ni HT Rock RB Peterson PK . WIN55, 212–2 inhibits production of CX3CL1 by human astrocytes: Involvement of p38 MAP kinase. J Neuroimmune Pharmacol. (2009) 4:244–8. doi: 10.1007/s11481-009-9147-5, PMID: 19214751 PMC2729711

[76] Hu B Wang Q Chen Y Du J Zhu X Lu Y . Neuroprotective effect of WIN 55, 212–2 pretreatment against focal cerebral ischemia through activation of extracellular signal-regulated kinases in rats. Eur J Pharmacol. (2010) 645:102–7. doi: 10.1016/j.ejphar.2010.07.024, PMID: 20667450

[77] Rabe A Wei Jie L Gurjar K Brackley A Ili D . Global burden of asthma, and its impact on specific subgroups: nasal polyps, allergic rhinitis, severe asthma, eosinophilic asthma. J Asthma Allergy. (2023) 16:1097–113. doi: 10.2147/JAA.S418145, PMID: 37822519 PMC10563777

[78] Webbeteg.hu . Napjaink népbetegsége: az allergiás nátha. Available online at: https://www.webbeteg.hu/cikkek/allergia/436/napjaink-nepbetegsege-az-allergias-natha (Accessed February 26, 2026).

